# Development of Mesoporous Silica Nanoparticle-Based Films with Tunable Arginine–Glycine–Aspartate Peptide Global Density and Clustering Levels to Study Stem Cell Adhesion and Differentiation

**DOI:** 10.1021/acsami.3c04249

**Published:** 2023-08-01

**Authors:** Xingzhen Zhang, Zeynep Karagöz, Sangita Swapnasrita, Pamela Habibovic, Aurélie Carlier, Sabine van Rijt

**Affiliations:** Department of Instructive Biomaterials Engineering MERLN Institute for Technology-Inspired Regenerative Medicine, Maastricht University, P.O. Box 616, 6200 MD Maastricht, The Netherlands

**Keywords:** global ligand density, ligand clustering level, biointerfaces, mesoporous silica nanoparticles, mesenchymal stem cell adhesion, RGD

## Abstract

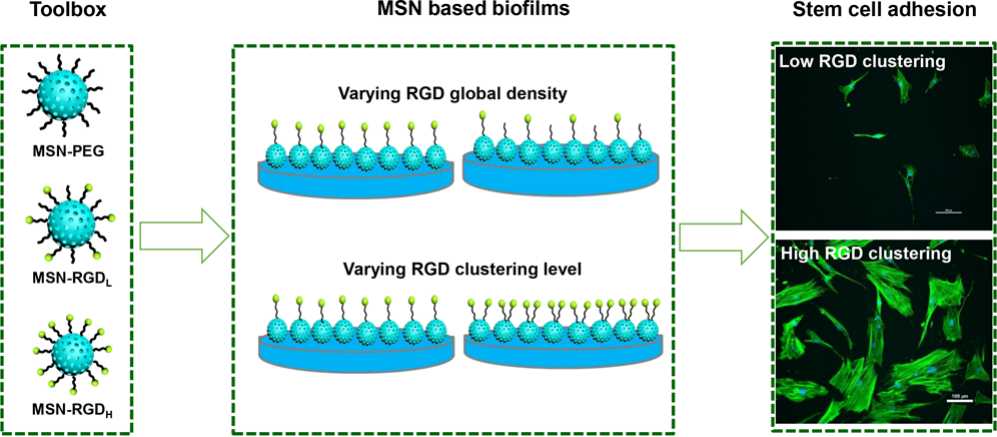

Stem cell adhesion is mediated via the binding of integrin receptors to adhesion motifs present in the extracellular matrix (ECM). The spatial organization of adhesion ligands plays an important role in stem cell integrin-mediated adhesion. In this study, we developed a series of biointerfaces using arginine–glycine–aspartate (RGD)-functionalized mesoporous silica nanoparticles (MSN-RGD) to study the effect of RGD adhesion ligand global density (ligand coverage over the surface), spacing, and RGD clustering levels on stem cell adhesion and differentiation. To prepare the biointerface, MSNs were chemically functionalized with RGD peptides via an antifouling poly(ethylene glycol) (PEG) linker. The RGD surface functionalization ratio could be controlled to create MSNs with high and low RGD ligand clustering levels. MSN films with varying RGD global densities could be created by blending different ratios of MSN-RGD and non-RGD-functionalized MSNs together. A computational simulation study was performed to analyze nanoparticle distribution and RGD spacing on the resulting surfaces to determine experimental conditions. Enhanced cell adhesion and spreading were observed when RGD global density increased from 1.06 to 5.32 nmol cm^–2^ using highly clustered RGD-MSN-based films. Higher RGD ligand clustering levels led to larger cell spreading and increased formation of focal adhesions. Moreover, a higher RGD ligand clustering level promoted the expression of alkaline phosphatase in hMSCs. Overall, these findings indicate that both RGD global density and clustering levels are crucial variables in regulating stem cell behaviors. This study provides important information about ligand–integrin interactions, which could be implemented into biomaterial design to achieve optimal performance of adhesive functional peptides.

## 1 Introduction

Stem cells are characterized by an inherent ability to self-renew and the potential to differentiate into specialized cells.^1^ In our body, stem cells play a vital role in tissue development, tissue homeostasis, and wound repair throughout life.^2,3^ Stem cell behavior such as self-renewal and differentiation is finely regulated by multifactorial cues provided by the extracellular matrix (ECM).^4^ The native ECM is an insoluble matrix containing intrinsic mechanical and biochemical cues that influence stem cell functions including adhesion, migration, and differentiation.^5,6^ In particular, the ECM provides instructive biochemical cues by presenting adhesive ligands, which are clustered and organized at the nanoscale and interact with stem cells to dictate cell behavior.^7^

Due to their inherent regenerative capabilities, stem cells have tremendous therapeutic potential for regenerating or repairing tissues and organs damaged by aging, cancer, and other diseases, and as such are intensively investigated in the regenerative medicine field.^8^ One of the major strategies in this field is to develop engineered biomaterials that can mimic the native ECM to elicit certain stem cell behavior.^9^ Ideally, the engineered biomaterials should not only provide essential structural and mechanical support but also contain biological and biochemical cues that can actively interact with cells to guide stem cell-mediated regenerative processes.^10,11^ In particular, designing bioactive materials able to drive specific cellular behaviors has been gaining more attention in the past few decades. One popular approach is to functionalize materials with tripeptide arginine–glycine–aspartate (RGD), which is a cell-adhesive ligand and can bind to integrin receptors on cellular membranes to enhance stem cell adhesion and integration with the materials.^12−14^ Integrin receptors are heterodimeric transmembrane proteins (containing α and β-integrin subunits) and are around 10 nm in size.^15,16^ The RGD sequence can bind to several different integrin dimers, i.e., αv β1, αv β3, αv β5, αv β6, αv β8, α5 β1, α8 β1, and αIIb β3, and is found in multiple ECM proteins such as fibronectin and vitronectin.^17^ Integrin-mediated cell focal adhesion (bundles of clustered integrins) and organization of cytoskeletal actin play a vital role in regulating various intracellular signaling pathways and subsequent cell properties.^18,19^ Hence, understanding integrin-mediated stem cell adhesion in the context of tissue regeneration is important in order to rationally design functional biomaterials able to control stem cell behavior.

Two-dimensional (2D) biointerfaces that offer high control over ligand presentation are popular material-based tools for studying receptor–ligand interactions.^20^ So far, numerous 2D biointerfaces have been created to study the effect of ligand-presenting patterns on integrin-mediated signaling.^21,22^ A traditional way to create a biointerface is to decorate a nonfouling surface with monovalent adhesive ligands, which randomly bind to integrin receptors. In this instance, control over the surface bioactivity can be achieved by tuning the ligand global density present at the interface.^23,24^ However, this random distribution of ligands only promotes integrin occupancy, not integrin clustering.^25^ Cell adhesion requires both integrin occupancy and integrin clustering.^26^ Integrin clustering is initiated by integrin dimerization and can be promoted by presenting ligands in a clustered format. Ligand clustering refers to incorporating multiple adhesive ligands within a small area and is an important factor that influences cell adhesion, spreading, and migration. For example, a surface with locally clustered ligands was generated by grouping several RGD ligands into isolated nanosized areas. It was shown that RGD ligand clustering could promote integrin clustering and facilitate the formation of adhesion complexes.^27^ While ligand clustering is known to enhance integrin activation, it is rare to investigate the effect of RGD global densities and clustering on stem cell behavior.^28−30^

In this study, we aimed to create a new type of biointerface, which allows us to control both the global density and local clustering level of RGD on the surface to a high extent. To create the biointerface, we propose a novel strategy based on mesoporous silica nanoparticles (MSNs). MSNs have been widely explored for various biomedical applications due to their favorable properties such as tunable morphology, good biocompatibility, porous structures, and easy surface functionalization. In addition, previously, we have shown that we could create homogenous and stable MSN films using a simple spin coating technique to specifically incorporate ligands onto the surfaces.^31^ Importantly, MSNs have a high surface area, which implies a high potential to graft densely clustered ligands at the nanoscale, and allows us to change the ligand clustering level with a high degree of control.^32^ Here, MSNs were functionalized with RGD peptides through a poly(ethylene glycol) (PEG) linker, which was used to resist protein absorption to the surface and prevent unspecific cell binding. Systematic variation in surface RGD global density has been achieved by blending different ratios of RGD-modified MSNs (MSN-RGD) with nonmodified MSNs (MSN-PEG) for spin coating, and the distribution of RGD on these resultant surfaces was analyzed by a computational simulation study. To vary the RGD ligand clustering level, MSN-RGD_H_ (high-clustered RGD) and MSN-RGD_L_ (low-clustered RGD) were synthesized. First, the effect of global ligand density on human mesenchymal stromal cell (hMSC) morphology and adhesion was studied. Then, we investigated the effect of the level of nanoclustered RGD on hMSC focal adhesion and differentiation.

## 2 Results

### 2.1 Synthesis and Characterization of MSN-PEG and MSN-RGD and Preparation of MSN Films

**Figure 1:**
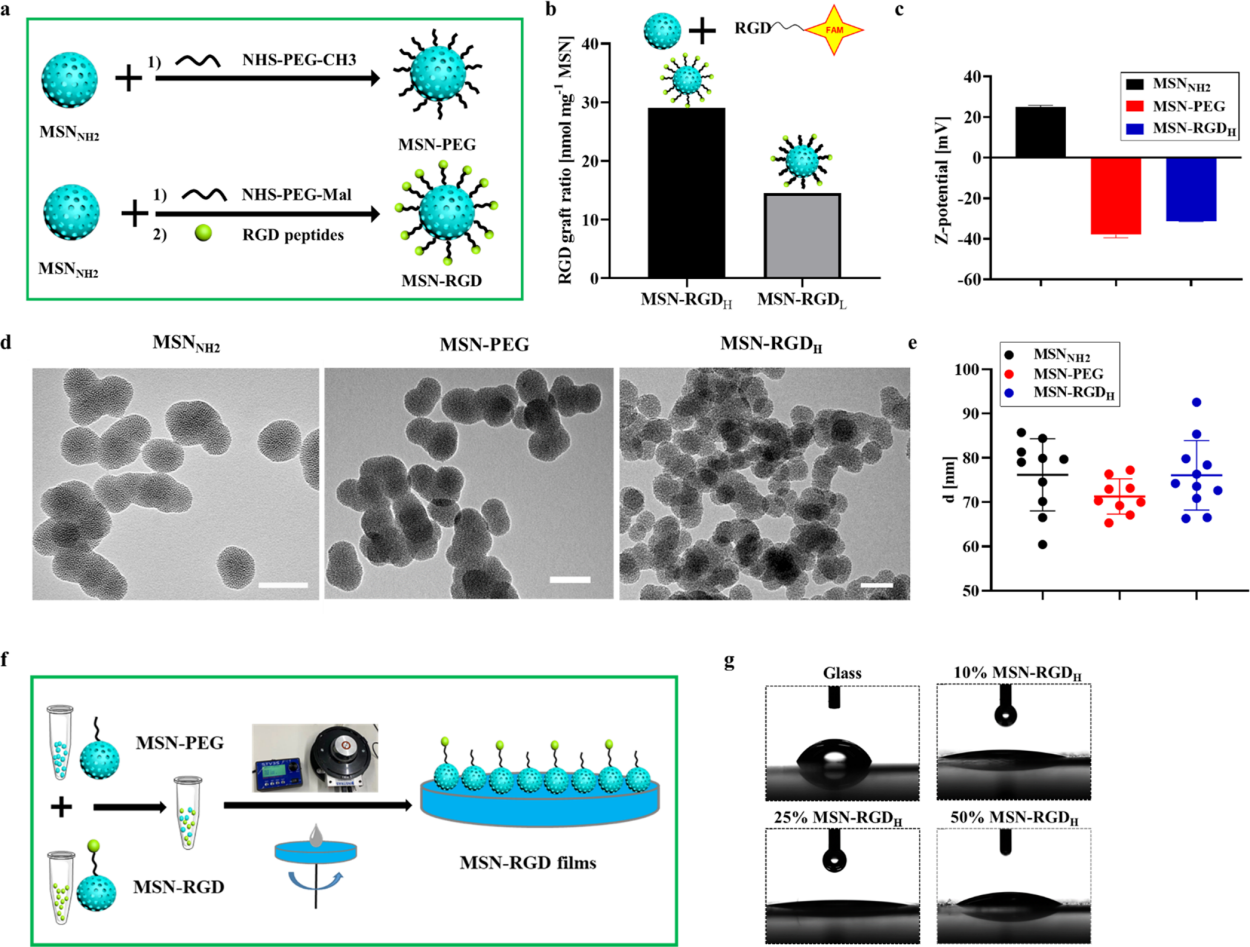
Synthesis and characterization of MSN-RGD. (a) Schematic illustration of surface functionalization of MSN_NH_2__ with PEG linkers and RGD peptides. (b) Quantification of RGD surface functionalization of MSN_NH_2__ using FAM fluorescently labeled RGD. (c) Zeta surface potential as measured by DLS in water showing the change in the surface charge after PEG and RGD surface functionalization. (d) TEM images of MSN_NH_2__, MSN-PEG, and MSN-RGD_H_ showing that as-synthesized nanoparticles have a spherical shape. The scale bar is 100 μm. (e) Particle size of MSN_NH_2__, MSN-PEG, and MSN-RGD_H_ as determined by TEM. (f) Schematic illustration of the preparation of MSN films with different surface RGD densities by spin coating of premixed nanoparticles. (g) Water contact angle of glass surfaces before and after coating with nanoparticles containing 10, 25, and 50% MSN-RGD_H_.

Synthesis of amine surface-functionalized mesoporous silica nanoparticles (MSN_NH_2__) was performed via hydrolysis and condensation of silica precursors in the presence of a micelle template, followed by surface grafting using 3-aminopropyl triethoxysilane (APTES), as we have reported recently.^33^ The presence of the amine group on MSNs was validated by labeling with 5/6-carboxyfluorescein succinimidyl ester (FITC-NHS, able to bind to amine groups). The fluorescent intensity of amine surface-modified MSNs was significantly higher compared to nonmodified MSNs (Figure S1). RGD functionalization was carried out via a two-step synthesis approach (Figure 1a). In the first step, the amine group was reacted with a heterobifunctional maleimide-PEG12-succinimidyl ester crosslinker (Mal-PEG12-NHS) to form MSN-PEG_mal_. Here, a PEG linker with 12 repeating units was selected as an antifouling spacer based on our earlier study showing that this length enables optimal RGD presentation.^31^ In the second step, a cysteine-modified RGD peptide was conjugated to MSN-PEG_mal_, yielding RGD-modified MSN (MSN-RGD). A FAM-tagged version of the RGD peptide (RGD-FAM) was used to monitor functionalization quantitatively (Figure 1b). By changing the RGD-FAM/MSN-PEG_mal_ ratios from 0.32 to 0.16 μmol mg^–1^, MSNs with high-clustered RGD (MSN-RGD_H_, 29.1 nmol mg^–1^) and low-clustered RGD (MSN-RGD_L_, 14.5 nmol mg^–1^) could be obtained, respectively (Figure 1b). MSN control nanoparticles functionalized with a PEG-CH_3_ linker (MSN-PEG) were synthesized by grafting a monofunctional m-dPEG-12-NHS ester linker to MSN_NH_2__ (Figure 1a).

Successful surface MSN amination and subsequent modification with PEG and RGD were further validated using ζ-potential measurements. MSN_NH_2__ had a positive surface charge of +24.9 mV due to the presence of an amine group on the surfaces, which became negative after PEG (−38.0 mV) and RGD modification (−31.4 mV) (Figure 1c). Transmission electron microscopy (TEM) was employed to characterize the nanoparticle shape and morphology and to monitor changes in size over the subsequent surface modifications with PEG and RGD. MSN_NH_2__ displayed an evenly round-shaped morphology with a uniform porous structure and had an average particle size of around 75 nm, as estimated from TEM images. The particle morphology and size were similar after surface modification with PEG and RGD (Figure 1d,e).

To create uniform films, concentrated nanoparticles were spin-coated over plasma-pretreated glass coverslips, as we reported previously.^34^ The global RGD density was varied by spin coating mixtures of 10, 25, and 50% MSN-RGD_H_ with MSN-PEG nanoparticles, which were designated as 10% MSN-RGD_H_, 25% MSN-RGD_H_ and 50% MSN-RGD_H_, respectively (Table 1). In addition, spin coating parameters including applied solvent and spin speed were optimized for the different nanoparticle compositions. An increased content of ethanol in the water solution resulted in improved coating homogeneity for MSN-RGD_H_ films (Figure S2a). As a result, an increased amount of ethanol was used as a solvent for spin-coating as the percentages of MSN-RGD increased. Uniform and homogenous films were obtained for all formulations (Figure S2b). The coating quality and surface roughness of MSN films were characterized using SEM and profilometer, respectively. The SEM images showed that a homogenous coating with continuous layers of nanoparticles spread over the glass substrate could be achieved (Figure S3). In addition, a three-dimensional (3D) laser scanning image of 50% MSN-RGD_H_ revealed a smooth surface profile (Figure S4a). The developed MSN films had a thickness of around 300 nm, indicating that the film was homogeneously covered and that 3–4 layers of nanoparticles were deposited over the glass substrate (Figure S4b). All MSN films showed a low surface roughness with a Ra of around 0.10 μm. No significant differences in roughness and thickness were found among MSN films that were made of different nanoparticle compositions (Figure S4c). The water contact angle (WCA) decreased from 60.5° for the glass surface to 20.5° after coating with 50% MSN-RGD_H_ (Figures 1g and S5), which further confirmed the successful creation of nanoparticle films. As surface wettability is known to influence cell adhesion^35^ and can be varied by surface chemical composition, we measured the WCA on the different MSN films. No significant difference in WCA was observed among surfaces prepared using 10, 25, and 50% MSN-RGD_H_ (Figure 1g).

**Table 1:** Weight % Composition of Nanoparticles Used for Preparation of MSN Films with Varying Global Densities and Clustering Levels

formulations	wt % MSN-RGD_H_	wt % MSN-RGD_L_	wt % MSN-PEG	RGD global density [nmol cm^–2^]
10% MSN-RGD_H_	10		90	1.06
25% MSN-RGD_H_	25		75	2.66
50% MSN-RGD_H_	50		50	5.32
50% MSN-RGD_L_		50	50	2.66
100% MSN-RGD_L_		100	0	5.32

### 2.2 Computational Calculation of RGD Distribution on MSN Films

**Figure 2:**
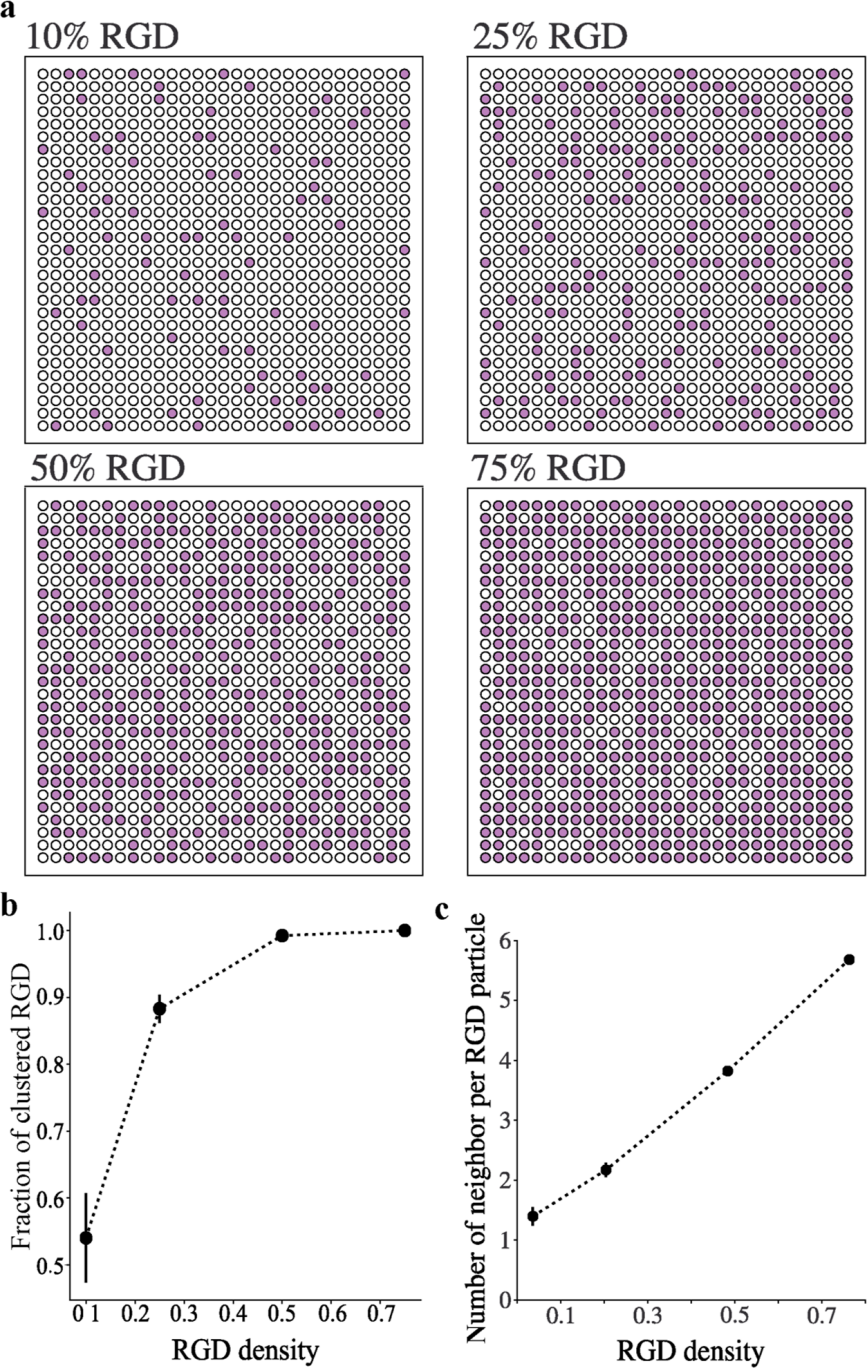
Computational calculation of spacing between adjacent MSN-RGD_H_ on a surface made from different ratios of MSN-RGD_H_ and MSN-PEG. (a) Computational description of how RGD clusters distributed over a surface made from varied MSN-RGD portions (Magenta circle, RGD-functionalized nanoparticles; white circle, non-RGD-containing nanoparticles). (b) Ratio of MSN-RGD particles that have at least one other MSN-RGD within a 70 nm distance over the total number of MSN-RGD particles on the surface. (c) Average number of neighboring MSN-RGD per nanoparticle on surfaces made from different weight percentages of MSN-RGD.

Computational simulations were performed to quantitatively assess nanoparticle distribution over the 2D surfaces to aid the selection of MSN-RGD variables for our experimental study. In these simulations, the random localization of the nanoparticles on the glass substrate was analyzed. We first made random distributions of 10, 25, 50, and 75% of MSN-RGD particles (MSN with a diameter of 70 nm and an RGD ligand attached to it) on a 2000 nm × 2000 nm grid (a 100% distribution implies 2000/70 × 2000/70 particles). We have also assumed mean-field approximation on the nanoparticle surface, i.e., the exact localization of the RGD ligand is not included in this study. A representative example of nanoparticles’ random distribution on surfaces is shown in Figure 2a. The pairwise distance between RGD ligands (note: the distance from surface to surface is only considered because of the mean-field approximation) was then calculated. For each MSN-RGD particle, only those MSN-RGD at a distance lower than 70 nm were defined as “neighbors.” Here, 70 nm was selected as a cutoff value because previous studies have shown that ligand spacing larger than ∼70 nm resulted in immature focal adhesions (because integrins cannot cluster), whereas ligand spacing smaller than ∼70 nm promoted maturation of focal adhesions.^36−40^ We replicated the steps (randomization of distribution, assigning neighbors) 100 times in order to remove removing any random number generator bias (Figure S6).^41^ First, the ratio of RGD particles that had at least one “neighbor” over the total number of MSN-RGD particles on the surface (Figure 2b) was calculated. Interestingly, this ratio was 100% on surfaces made from 50% MSN-RGD, which meant every RGD-modified nanoparticle had at least one neighbor of MSN-RGD within a 70 nm distance on the surface. However, the ratio for surfaces prepared with 10% RGD-modified nanoparticles to have one “neighbor” is as high as 50% (probability(RGD particle with RGD neighbor) = 1 – probability(having no RGD neighbors) = 1 – (0.9)^8^ = 0.57). To study the effect of the nanoparticle size on RGD ligand distribution at a certain RGD global density (25% RGD), we varied the diameter of nanoparticles (50, 70, 90, and 120 nm) and recalculated the ratio of “clustered RGD” over the total number of MSN-RGD particles. This ratio decreased with a larger nanoparticle diameter (Figure S7). When the diameter was 50 nm, there were 12 “neighbors” that were within the cutoff distance of 70 nm at a global density of 25%, which gave a probability of 1 – (0.75)^20^ = ∼0.9968. As the diameter increased to 120 nm, the number of “neighbors” within the 70 nm cutoff distance decreased to 8, and the chance of having at least one neighbor in the 8 neighbors = 1 – (0.75)^8^ = ∼0.89. Additionally, we also plotted the average number of RGD “neighbors” of the RGD peptide-containing particles, which had at least one other MSN-RGD within a 70 nm distance over the total number of MSN-RGD particles based on 100 different surface coatings (Figure 2c) to calculate average aggregate size. On average, on 10% MSN-RGD surfaces, the MSN-RGD aggregate consisted of 2 RGD particles, while the aggregate size of 50% MSN-RGD surfaces is approximately 5. Overall, as the fraction of RGD particles increased, the ratio of RGD particles that had at least one “neighbor” increased, and at a limit of 50% RGD particles, the neighboring ratio reached a plateau of 100% with an aggregate size of 5.^42^ Therefore, 10% MSN-RGD, 25% MSN-RGD, and 50% MSN-RGD surfaces were selected as our experimental groups for the cell adhesion study.

### 2.3 Effect of RGD Global Density on hMSC Morphology and Spreading of High-Clustered MSN-RGD Films

**Figure 3:**
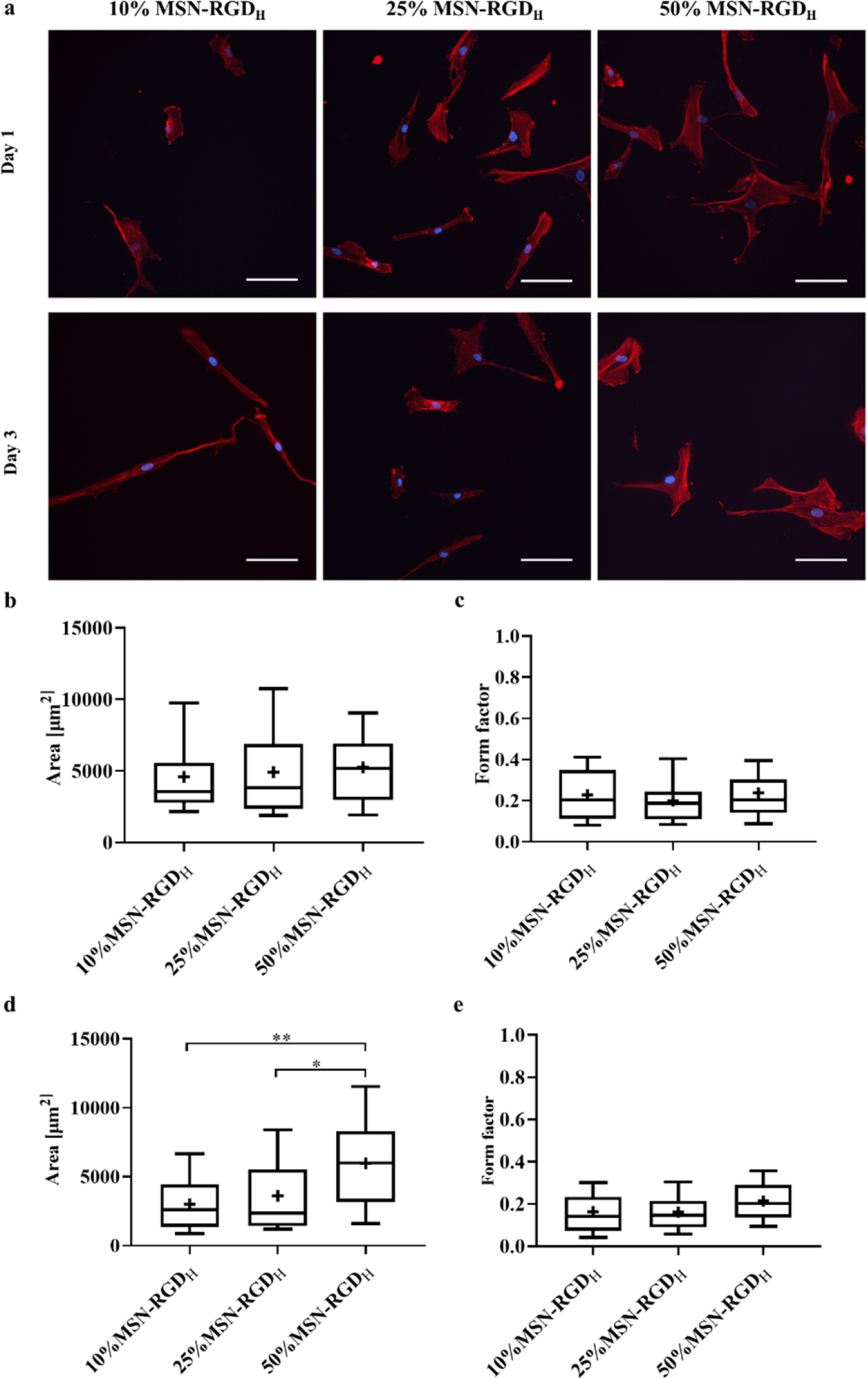
Effect of the global ligand density on hMSC morphology. (a) Representative fluorescent microscopy images showing the distinct morphology of hMSCs grown on different MSN films composed of 10, 25, and 50% of MSN-RGD_H_ for 1 day and 3 days. Cells were stained to visualize F-actins (red) and nuclei (blue). The scale bar is 100 μm. Box plots showing the (b) cell area and (c) form factor of hMSCs grown on three different MSN films for 1 day; (d) Cell area and (e) form factor after 3 days of culture on films containing 10, 25, and 50% MSN-RGD_H_. Data are shown as the mean ± SD from 4 biological replicates. *p < 0.05; **p < 0.01; ***p < 0.001.

Cells need enough adhesion sites with defined interligand spacing to be able to adhere. Several studies have highlighted the importance of ligand spacing on integrin-mediated cell adhesion processes.^43−45^ However, very few studies report on the ligand global density range that is required for stem cell adhesion. Here, the effect of RGD global density (RGD coverage over the surface) on hMSC spreading and morphology was assessed by seeding hMSCs on MSN films containing 10, 25, and 50% MSN-RGD_H_. After 1 and 3 days, hMSCs were stained to visualize the nuclei and cytoskeletal F-action organization and imaged using fluorescence microscopy (Figure 3a). hMSCs cultured on the 50% MSN-RGD_H_ surface (high global RGD density of 5.32 nmol cm^–2^) showed spread morphology with well-defined stress fibers (Figure 3a). In contrast, cells cultured on a 10% MSN-RGD_H_ surface (low global RGD density of 1.06 nmol cm^–2^) were only stretched in one or two directions with limited formation of stress fibers. The attached cell number, cell spreading area, and form factor (form factor approaches 1 for highly circular cells) of adhered hMSCs were further analyzed using Cell Profiler. After 1 day of culture, the cell spreading area and the number of adhered hMSCs showed an increasing trend as RGD global density increased (Figures 3b and S8a). After 3 days of culture, cells cultured on 50% MSN-RGD_H_ surfaces had significantly higher cell area compared to hMSCs cultured on 10 and 25% MSN-RGD_H_ surfaces (Figure 3d). Moreover, the total cell numbers present on 50% MSN-RGD_H_ surfaces were significantly higher compared to the other two MSN films (Figure S8b). No significant differences in the form factor were observed for hMSCs cultured on the three different surfaces after 1 day of culture (Figure 3c). However, there was an increase in elongation of the cells (smaller form factor) when cultured on 10% MSN-RGD_H_ surfaces, as compared to hMSCs cultured on surfaces with 50% MSN-RGD_H_ after 3 days of culture (Figure 3e).

### 2.4 Effect of Nanoscale RGD Ligand Clustering Levels on hMSC Morphology and Adhesion at Varied RGD Global Densities

**Figure 4:**
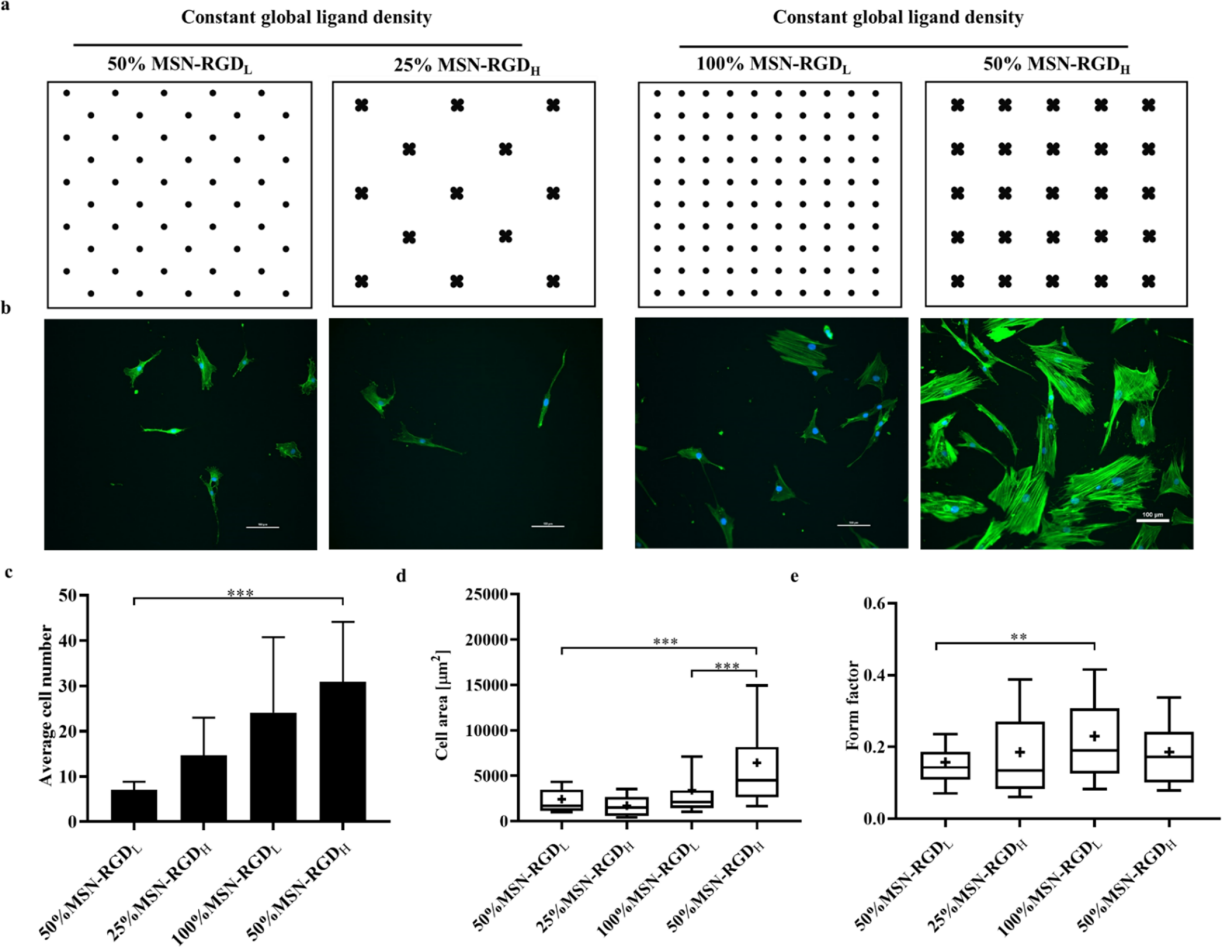
Effect of RGD global density and clustering on hMSC morphology. (a) Schematic illustration of the developed MSN films showing RGD local and global density. (b) Morphology of hMSCs adherent to MSN films with different nanoscale RGD ligand clustering while maintaining the same global RGD density after 3 days of culture. Cells stained for nuclei (DAPI; blue) and actin (phalloidin 488; green). The scale bar is 100 μm. (c) Attached cell number on highly clustered films compared to low-clustered films. Box plots showing the (d) cell area and (e) form factor of hMSCs grown on four different MSN films for 3 days. Data are expressed as the mean ± SD (*n* = 18–97, 3–6 images from biological triplicates). *p < 0.05; **p < 0.01; ***p < 0.001.

Next, we studied the effect of RGD ligand clustering levels at different global ligand densities on hMSC morphology and spreading. While maintaining the global RGD density constant, two surfaces with varied RGD clustering levels were generated by using MSN-RGD_H_ or MSN-RGD_L_ blended together with MSN-PEG nanoparticles. Specifically, two types of MSN films were developed with the same global density of 2.66 nmol cm^–2^, which consisted of 50% MSN-RGD_L_ mixed with 50% MSN-PEG and 25% MSN-RGD_H_ mixed with 75% MSN-PEG. A second set of MSN films were created that contained 5.32 nmol cm^–2^ RGD global density, which consisted of 50% MSN-RGD_H_ mixed with 50% MSN-PEG and 100% MSN-RGD_L_. A schematic illustration of the RGD-nanoparticle distribution of the prepared surfaces is shown in Figure 4a. hMSCs were cultured on the four different MSN films for 3 days and then stained for filamentous actins (F-actins, green) and nuclei (blue). A distinct difference in the morphology of hMSCs was observed between 100% MSN-RGD_L_ and 50% MSN-RGD_H_ (Figure 4b). Overall, hMSCs adhered to surfaces with high global RGD density (100% MSN-RGD_L_ and 50% MSN-RGD_H_) showed better-organized actin assembly and spreading morphology compared to cells cultured on surfaces with low global RGD density (50% MSN-RGD_L_ and 25% MSN-RGD_H_), which had an elongated cell shape. Interestingly, although a similar cell shape was observed on 50% MSN-RGD_H_ and 100% MSN-RGD_L_ surfaces, cells grown on the highly clustered RGD surface (50% MSN-RGD_H_) were much larger in comparison to that on the 100% MSN-RGD_L_ surface (Figure 4b).

Single cells were outlined using Cell Profiler software to calculate cell spreading areas and form factors. The attached cell number of hMSCs cultured on highly clustered RGD surfaces (50% MSN-RGD_H_) was significantly higher compared to hMSCs adhered to low-clustered RGD surfaces (50% MSN-RGD_L_) (Figure 4c). In addition, hMSCs spread out more when adhered on 50% MSN-RGD_H_ than on 50% MSN-RGD_L_ and 100% MSN-RGD_L_ surfaces (Figure 4d). There was a significant difference in cell spreading between 50% MSN-RGD_H_ and 100% MSN-RGD_L_ surfaces, suggesting a clustering effect on cell spreading. However, this difference was not observed when comparing cell spreading on 25% MSN-RGD_H_ and 50% MSN-RGD_L_ surfaces. Together, these findings suggests that a local ligand clustering level below a 70 nm scale has an effect on cell morphology and spreading, and that this effect is also global ligand density-dependent.

**Figure 5:**
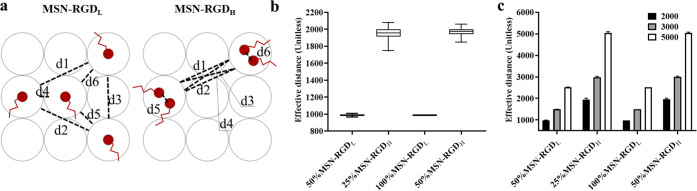
Characterization of the effective distance. (a) Schematic presentation of the calculation of the effective distance on high- and low-clustered nanoparticle surfaces. In a low-local-density (MSN-RGD_L_) situation, the effective distance between the RGD particles is the average of all distances and is calculated as (*d*1 + *d*2 + *d*3 + *d*4 + *d*5 + *d*6)/6. In a high-local-density (MSN-RGD_H_) situation, the effective distance becomes (*d*1 + *d*2 + *d*3 + *d*4 + *d*5 + *d*6)/6. We neglect *d*5 and *d*6 because of the mean-field approximation. We can also approximate *d*1 = *d*2 = *d*3 = *d*4. So the effective distance becomes 4* *d*1/4 = *d*1. (b) Effective distance between MSN-RGD particles on surfaces with varied global and local densities. (c) Effective distance on surfaces with varied surface sizes.

Next, we calculated the effective distance on clustered surfaces to help explain our observations of cell morphology and spreading. The effective distance in this study was defined as the average ligand distance between any two RGD particles and was calculated by the sum of distances between any two RGD nanoparticles divided by the total number of RGD nanoparticles on the substrates (Figure 5a). A higher effective distance would imply a higher ligand interaction and, as a result, higher cell spreading. When we simulated the coating on a surface with a size of 2000 × 2000 (unitless dimension), we observed a higher effective distance for high-local-density particles (MSN-RGD_H_) than MSN-RGD_L_, and this pattern did not change with an altered global density of RGD (Figure 5b). To make sure our observation was not affected by the size of the surface we chose, we also studied the effect of surface size on the effective distance. We found that irrespective of the surface size, high-local-clustering particles always had a larger effective distance (Figure 5c). This simulation also explained our experimental observations, where an increased cell spreading area of 50% MSN-RGD_H_ in comparison to 100% MSN-RGD_L_ could be attributed to a higher effective distance of high-RGD-clustered particle-coated surfaces.

### 2.5 Focal Adhesion

**Figure 6:**
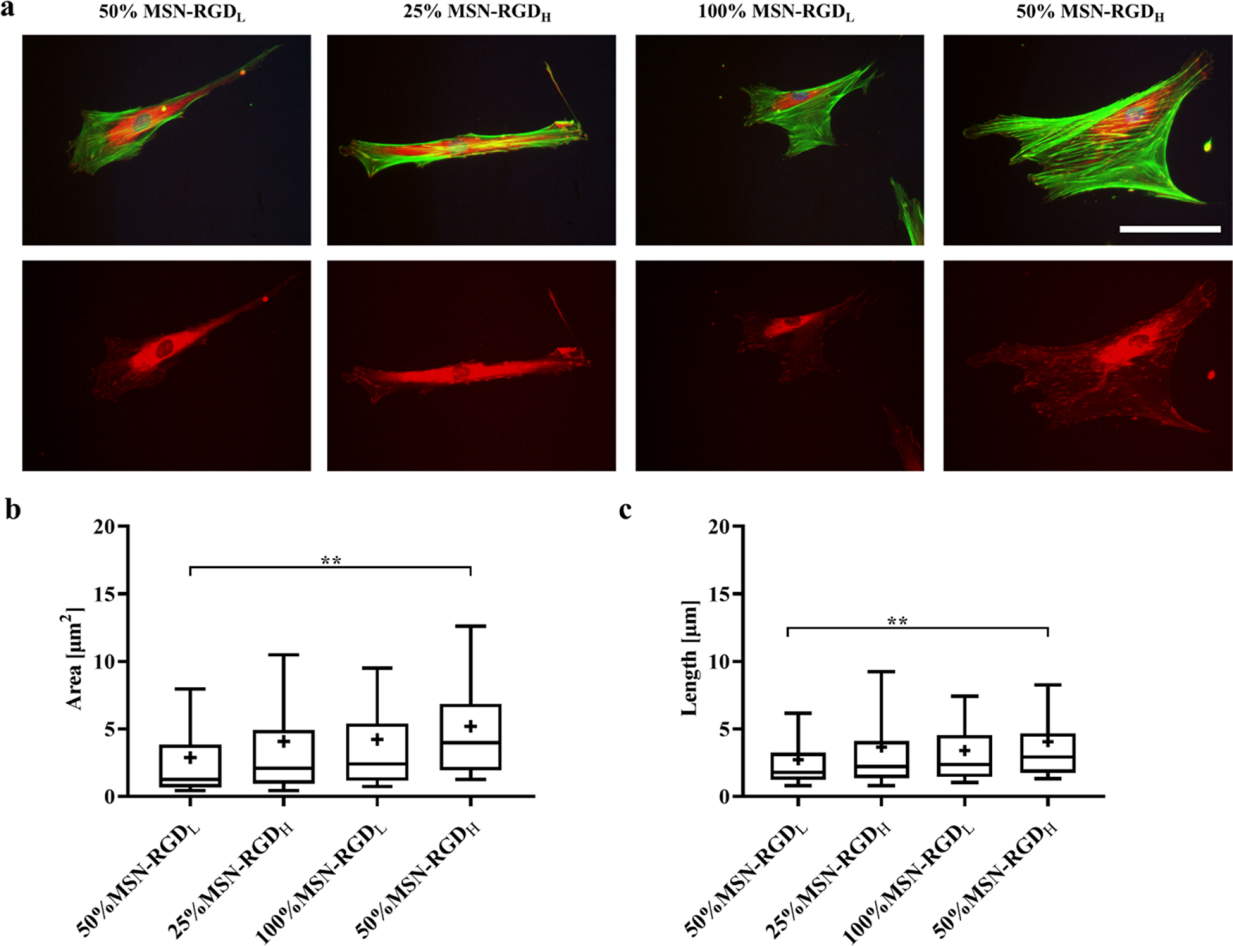
Effect of nanoscale ligand clustering levels on hMSCs focal adhesion points. (a) Representative fluorescence microscopy images of hMSCs cultured on MSN films showing focal adhesions after 5 days of culture. hMSCs were stained for focal adhesion protein vinculin (red), actin (green), and nuclei (blue). The vinculin staining (red) was shown separately. The scale bar represents 100 μm and applies to all images. (b) Area and (c) length of focal adhesions after 5 days. Data are presented as the mean ± SD (*n* = 89–104 from biological triplicates). *p < 0.05; **p < 0.01; ***p < 0.001.

Focal adhesions (FAs) are key for cell anchorage and organization of the actin cytoskeleton.^46^ Thus, we next studied how ligand clustering levels at varied global ligand densities influence the vinculin expression of hMSCs, which is a protein recruited from the cytoplasm to the focal adhesion complex.^47,48^ hMSCs were cultured on 100% MSN-RGD_L_, 50% MSN-RGD_H_, 50% MSN-RGD_L_, and 25% MSN-RGD_H_ surfaces for 5 days and then stained for vinculin (red), F-actin bundles (green), and nuclei (blue). In accordance with our previous observations, a more spread morphology with prominent actin cytoskeleton alignment could be observed for hMSCs cultured on 50% MSN-RGD_H_ (Figure 6a). Additionally, hMSCs on the 50% MSN-RGD_H_ substrates exhibited a higher vinculin expression, with larger and longer focal points formed compared to cells cultured on 100% MSN-RGD_L_ substrates (Figure 6a–c), suggesting that the presentation of RGD in a highly clustered format resulted in more efficient grouping of integrin receptors as compared to the same surface global density of RGD but with a low clustering level.

### 2.6 Effect of RGD Ligand Clustering on ALP Production in hMSCs

**Figure 7:**
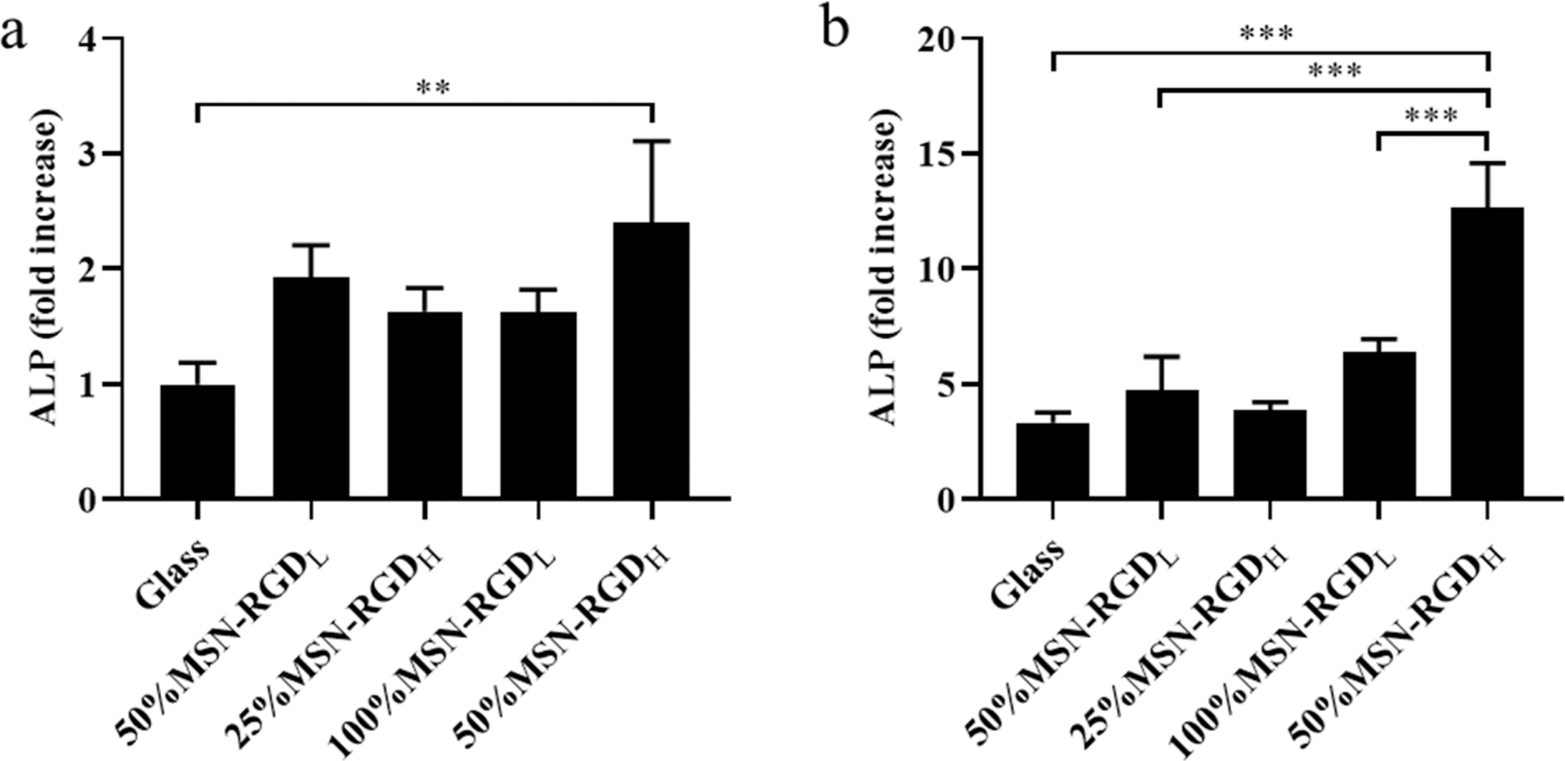
Effect of RGD ligand clustering and local density on hMSC ALP production. ALP production of MSCs cultured on the four MSN films and glass for 14 days in (a) basic medium and (b) osteogenic medium. hMSCs cultured on glass in basic medium were the negative control. Values are *x*-fold increases compared to the negative control. Data are shown as the mean ± SD of biological triplicates. *p < 0.05; **p < 0.01; ***p < 0.001.

Cytoskeleton organization, cell spreading, and focal adhesion is known to influence stem cell differentiation.^49^ To test if the difference in the initial cell focal adhesion on our biointerfaces could induce cell differentiation processes, hMSCs were cultured on 100% MSN-RGD_L_, 50% MSN-RGD_H_, 50% MSN-RGD_L_, and 25% MSN-RGD_H_ surfaces either in basic or osteogenic medium (with 10 nM Dex supplementation) for 14 days. After 14 days of cell culture, alkaline phosphatase (ALP) production in hMSCs was examined. ALP is one of the earliest markers of osteogenic differentiation and has been widely used for evaluating the osteogenic potential of hMSCs.^50^ hMSCs cultured on glass slides in basic medium were used as a control. In basic conditions, significantly higher ALP levels were observed for hMSCs cultured on 50% MSN-RGD_H_ films compared to negative control conditions (Figure 7a), indicating that RGD ligand clustering at high RGD global density can promote osteogenic marker expression when no other osteogenic stimulants are present. Interestingly, this was not observed for lower clustered RGD surfaces with the same RGD density (100%MSN-RGD_L_). Furthermore, this effect was more pronounced when hMSCs were cultured in osteogenic medium; ALP expression of the 50% MSN-RGD_H_ film was 12.4-fold compared to a 3.3-fold ALP increase for cells cultured on glass controls (Figure 7b). Remarkably, hMSC cultured on surfaces that contained the same RGD density but had lower RGD clustering (i.e., 100% MSN-RGD_L_) did not show significantly increased ALP production compared to glass controls. These data indicate that RGD ligand clustering at high RGD density can play an important role in promoting osteogenic differentiation in hMSCs.

## 3 Discussion

In this study, we presented a novel strategy to create biointerfaces based on RGD-modified silica nanoparticles, which enables high control over the RGD ligand clustering level and global density of 2D surfaces. Although several studies report on the importance of ligand clustering to promote (stem) cell adhesion processes, no previous study has looked into the effect of the ligand clustering level on integrin-mediated adhesion and stem cell function. We fabricated four MSN films using high- and low-clustered RGD on MSN surfaces to investigate the effect of the RGD nanoscale clustering level and spacing on hMSC adhesion and differentiation. We found that the RGD ligand clustering level regulates integrin-mediated stem cell adhesion and ALP expression.

In our approach, we used MSNs to create a novel biointerface capable of presenting clustered ligands in a controlled way. MSN was successfully functionalized with RGD peptides using a PEG linker. Here, a PEG linker was used as an antifouling spacer to resist nonspecific protein adsorption and cell adhesion. As the PEG chain length could potentially influence its antifouling property and ligand functionality, it is also important to optimize the PEG spacer length in any system to ensure proper cell adhesion. In this work, PEG12 was selected as an antifouling spacer based on our previous study, where we showed that this PEG length was optimal for studying specific hMSC-RGD interactions.^31^ Our approach enabled tunable RGD functionalization by changing the ratios of MSN to RGD during synthesis. Using this method, we could control the RGD ligand clustering level on the silica nanoparticles to create high- and low-RGD-clustered MSNs. Considering the large surface area and nanometer size of MSN, our platform allows tailoring of the RGD ligand clustering level at the nanoscale. Indeed, our results showed that we can achieve a higher RGD ligand clustering level of 29.1 nmol mg^–1^ compared to other substrates reported in the literature.^27,51^ In addition, the particle size can be easily tuned, which therefore enables precise control over the size of ligand islands. Another advantage of our novel interface is the simplicity of the preparation process. Previously, ligand immobilization and patterning on surfaces have been achieved using several distinct methods such as the nanolithography technique and covalent surface grafting, which either relies on expensive and specialized equipment or involves aggressive and complex fabrication processes.^25^ For example, traditional covalent immobilization of ligands to glass substrates often required piranha treatment to activate the hydroxyl groups of glass surfaces.^52,53^ In our approach, using a simple blending and spin coating technique, homogeneous MSN films made from different nanoparticle compositions could be created and therefore ensure easy modulation of ligand distribution.

To investigate what RGD global density range was required to stimulate cell adhesion when the ligands were highly clustered at the nanoscale, three MSN films with a varied RGD global density were generated. We observed enhanced cell adhesion and spreading as global RGD density increased. Specifically, hMSCs on high-RGD-density surfaces presented a more spread morphology with a better-organized actin cytoskeleton as compared to that on low-RGD-density surfaces. Cytoskeletal components such as actin have been reported to regulate stem cell differentiation with a higher cytoskeleton tension, leading to greater osteogenic differentiation.^54−56^ The increase of cell adhesion with the increase of RGD density is consistent with a previous study reporting cell adhesion of another cell type (C2C12 skeletal myoblasts).^57^ However, the surface RGD density that can induce proper cell adhesion in our system was 5.32 nmol cm^–2^, which is much lower compared to earlier published data of 1.2 × 10^3^ nmol cm^–2^.^58^ This difference could be, in part, explained by the high clustering of RGD ligands at the nanoscale in our system. Previously it has been shown that the presentation of integrin-binding ligands in a clustered format resulted in enhanced integrin clustering and the formation of focal complexes.^59^ Furthermore, the different cell types could be another explanation, as it has been previously reported that the effect of local and global ligand density is distinct for different cell types.^25^

To investigate the effect of the RGD ligand clustering level on stem cell adhesion, we used MSN with varied RGD ligand clustering levels to create four different MSN films. We observed that when we kept global ligand densities the same, surfaces with more highly clustered RGD promoted cell adhesion. Specifically, highly clustered surfaces resulted in increased cell numbers, larger cell spreading, and larger and longer focal adhesion points as compared to lower RGD clustered surfaces. The binding of integrins to ECM ligands induces a conformational change in the structure of the cytoplasmic tail of the integrin, which initiates integrin clustering and subsequent formation of focal complexes.^59^ As such, the enhanced cell adhesion to the highly clustered RGD surface may be related to a higher level of integrin clustering that was mediated by the clustered RGD ligands.^60^ The positive effect of ligand clustering on cell adhesion has also been reported previously.^61^ In this study it was shown that at the maximal reported density of 30,000 YGRGD ligands per square micrometer, the cell response was significantly lower when exposed to individual YGRGDs compared to cells exposed to ligands clustered in groups of nine and with a cluster density of only 2300 YGRGD μm^–2^. However, in our study, the clustering effect was not observed when lower global RGD densities were used; here, the clustering level did not show a significant effect on cell number and spreading. This is likely due to insufficient adhesive binding sites and no integrin clustering at low-global-density surfaces, which could potentially lead to low cell adhesion, cell quiescence, or even apoptosis.^62^ Similar to our findings, Benitez et al. also reported that ligand clustering influences integrin-dependent signals in a manner that significantly depends on both global and local ligand densities.^63^

The clustering level also influenced the ALP activity of hMSCs, where highly clustered surfaces could significantly upregulate ALP levels in hMSCs compared to low-clustered surfaces. The difference in ALP production was well-aligned with the differences we observed in hMSC morphology and spreading. It can then be speculated that cells with more spread morphology and stronger adhesions undergo osteogenic differentiation. This result aligns with previously published data, in which larger and increased numbers of focal adhesions formed on smaller nanospacing-promoted higher levels of mechanical tension and, therefore, biased the commitment of hMSCs to an osteogenic fate through enhanced mechanotransduction.^64^ Indeed, previous studies have reported that focal adhesions emerge as diverse protein networks which not only provide structural integrity connecting the ECM to the intracellular actin cytoskeleton but also transmit signaling pathways crucial to cell differentiation.^65^ The exact signaling mechanisms linking focal adhesions with the commitment of hMSCs to the osteogenic lineage are still not well understood. However, several studies have suggested that the FAK → ERK → Runx2 signaling pathway constitutes a crucial element of the transduction machinery controlling this process.^66,67^ In summary, our findings suggest that the RGD ligand clustering level also had an effect on hMSC adhesion and differentiation, and that the effects of RGD ligand clustering are dependent on global ligand density.

## 4 Conclusions

In conclusion, we fabricated a series of biointerfaces based on RGD-modified MSN to study the effect of the RGD global density and nanoscale clustering level on stem cell morphology, focal adhesion, and differentiation. Distinct differences in hMSC morphology and spreading were observed as the average global RGD density changed. The nanoscale RGD ligand clustering level could be tuned and a higher RGD ligand clustering level led to an enhanced focal adhesion and osteogenic differentiation even when the global RGD density remained consistent. This suggested that the nanoscale ligand clustering level could be a crucial factor to be considered to optimize RGD incorporation into biomaterials. Our findings highlight the importance of nanoscale ligand clustering in biomaterial design in the regulation of stem cell response. Ligand clustering could be more beneficial to enhance cell adhesion than randomly increasing ligand density. We expect the knowledge gained from this study to accelerate the development of more functional materials to support stem cell-based regenerative therapies.

For future applications, the fabricated MSN-RGD platform is not limited to the study of the RGD-integrin interaction but also allows the incorporation of other ligands to probe other ligand-induced stem cell processes. The possibility to tune the surface chemistry of MSN makes them versatile platforms that may be engineered to display multiple epitopes to study nanoscale ligand crosstalk. Moreover, our MSN with clustered RGD can also be easily incorporated into biomaterials to enhance their (stem) cell adhesion properties and/or improve tissue integration.

## 5 Experimental Section

### 5.1 Reagents

Tetraethyl orthosilicate (TEOS, 98%), triethanolamine (TEA), cetyltrimethylammonium chloride (CTAC), 3-aminopropyl triethoxysilane (APTES), ammonium fluoride, hydrochloric acid (37%), m-dPEG12-NHS ester (PEG-CH_3_), ammonium nitrate, phosphate-buffered saline (PBS), fetal bovine serum (FBS), and ascorbic acid 2-phosphate sesquimagnesium salt hydrate (ASAP) were purchased from Sigma-Aldrich. RGDC peptides were purchased from Sanbio. RGD-FAM was commercially synthesized by GenScript. Absolute ethanol, paraformaldehyde (PFA), minimum essential medium α GlutaMAX (αMEM), bovine serum albumin (BSA), and Triton X-100 were purchased from VWR. 5/6-carboxyfluorescein succinimidyl ester (FITC-NHS), CyQUANT cell proliferation assay kit, and Mal-PEG12-NHS were purchased from ThermoFisher Scientific. The alkaline phosphatase (ALP) assay kit and recombinant Alexa Fluor 647 anti-vinculin antibody were purchased from Abcam. Penicillin and streptomycin were obtained from Gibco Life Technologies. Alexa Fluor 488 Phalloidin and Alexa Fluor 647 Phalloidin were purchased from Fisher Scientific.

### 5.2 Synthesis and Characterization of MSN_NH_2__ MSN-PEG and MSN-RGD

Synthesis of MSN_NH_2__ was based on a sol–gel co-condensation process, as previously reported.^68^ Further details on MSN_NH_2__ synthesis and characterization can be found in the Supporting Information.

Conjugation of RGD onto MSN_NH_2__ was performed in two steps. First, MSN_NH_2__ was modified with an NHS-PEG12-Mal linker to create MSN-PEG_mal_. For this, 2 mg of MSN_NH_2__ was dispersed in 920 μL of PBS buffer (pH 8.25) and sonicated for 30 min at room temperature (RT). Then, 80 μL of Mal-PEG12-NHS (5 mM in DMSO) was added and the mixture was stirred for 4 h. After that, MSN-PEG_mal_ was obtained by centrifugation and purified by subsequent washing with water. In the second step, the obtained MSN-PEG_mal_ was redispersed in 600 μL of Tris-EDTA buffer (pH 7.4), followed by the addition of 400 or 200 μL of RGDC peptides (2 mg/mL in water) to create MSN with high-clustered or low-clustered RGD, respectively. Then, the RGD coupling reaction was carried out by continuously stirring the mixture overnight at RT. Finally, MSN-RGD were collected by centrifugation, followed by washing, and then stored at 4 °C.

To quantify the RGD grating ratio, FAM-labeled RGD peptides (RGD-FAM) were used for the reaction as described above instead of using RGDC peptides. After the RGD coupling reaction, unbound RGD-FAM peptides were collected and quantitatively calculated by fluorescence intensity measurements at λ_ex_ = 488 ± 14 nm and λ_em_ = 535 ± 30 nm. A standard curve prepared from RGD-FAM was used for calibration.

MSN-PEG was also created and used as a blank control in this study. For this, appropriate amounts of the m-dPEG12-NHS ester linker (5 mM in DMSO) were added to the MSN_NH_2__ suspension and stirred for 4 h. After that, excess linkers were removed by double-washing in water. MSN-PEG was collected by centrifugation and stored at 4 °C.

The ζ-potential of MSN_NH_2__, MSN-PEG, and MSN-RGD_H_ was analyzed using a Malvern Zetasizer Nano (Malvern Panalytical, U.K.). For this, nanoparticles were suspended in Milli-Q water at 0.5 mg/mL concentration and sonicated for 30 min. The morphology and size of MSN_NH_2__, MSN-PEG, and MSN-RGD_H_ were examined using transmission electron microscopy (TEM, JEM-100CX II, Japan). Nanoparticles that were suspended in absolute ethanol at 0.5 mg/mL concentration were dropped onto a copper grid and air-dried at RT overnight before imaging. Particle size was determined using ImageJ.

### 5.3 Preparation and Characterization of MSN-Based Films

MSN films were prepared using spin coating. Immediately prior to spinning, coverslips with a diameter of 22 mm were surface-cleaned in 1 M HCl in 50% ethanol and activated with O_2_ plasma treatment (Plasma Cleaner, Diener Electronics Femto PCCE) at 0.4 bar, 5 sscm O_2_, 70 W, 10 min. MSN-PEG was collected by centrifugation (14000 rpm, 20 °C, 10 min) and dispersed in bidistilled water at a concentration of 40 mg/mL. Various ethanol–water solvents including 0, 50, and 70% of ethanol have been used to disperse MSN-RGD to optimize nanoparticle dispersibility and spin coating homogeneity. To prepare MSN films with various global RGD densities at high clustering levels, different ratios of MSN-PEG and the MSN-RGD_H_ dispersant were mixed accordingly and a volume of 25 μL of the mixture was pipetted centrally on a coverslip and spun at 2100 rpm for 20 s on a tabletop spin coater. Similarly, MSN films with low clustering levels were created by mixing an appropriate proportion of MSN-RGD_L_ with MSN-PEG for spin coating. The obtained films were stored dry at 4 °C. The spin coating quality and film homogeneity were assessed by optical pictures. To further characterize the films, 3D laser scanning microscopy (Keyence VR-3000 3D Profilometer, Keyence, Japan) was used to assess film roughness and thickness. SEM (Teneo, FEI) imaging was used to analyze the surface properties of the films and assess the coating homogeneity. For SEM analysis, spin-coated MSN films were sputtered with a 2 nm layer of iridium and imaged at 25,000 × and 10,000 × magnification. WCA of MSN films was measured by a sessile drop technique at room temperature using a contact angle goniometer (Drop shape Analyzer DSA25, Kruss, Germany). For this, nanoparticle spin-coated coverslips and uncoated coverslips were fixed on a stage of the goniometer. A 5 μL droplet of water was dropped onto the films and the values were read after 1 min.

### 5.4 hMSC In Vitro Cell Culture

hMSCs were obtained from one donor with informed consent and cultured in αMEM medium with the addition of 10% (v/v) FBS, 0.2 mM ASAP at 37 °C, and 5% CO_2_ in a humidified atmosphere. Cells before passage 6 were used for the experiments. Cell seeding densities varied depending on the individual experiment, and detailed information can be found in the Experimental Section.

### 5.5 Cell Morphology on Films and Image Analysis

Cell morphology on MSN films was evaluated by staining hMSCs for F-actin and nuclei using Alexa Fluor 647 Phalloidin and 4′,6-diamidino-2-phenylindole (DAPI). hMSCs were seeded onto the films at a density of 3000 cells cm^–2^ and 1000 cells cm^–2^ for culturing 1 day and 3 days, respectively. Before staining, cells were rinsed with PBS and fixed with 4% PFA for 15 min. After three times of washing with PBS, samples were incubated with freshly prepared Triton X-100 (0.2% (vol/vol) in PBS) for 10 min and blocked with blocking buffer (4% (w/v) BSA and 0.05% (v/v) Tween in PBS) for 1 h at RT. After blocking, the cells were stained with Alexa Fluor 647 Phalloidin (1:40 in PBS) overnight at 4 °C, followed by DAPI staining (1:100 in PBS) for 15 min. Then, the films were rinsed with PBS, mounted on a glass slide with mounting media (Dako), and imaged using a Nikon Eclipse Ti-E microscope (Nikon Instruments Europe BV, the Netherlands) at 20× objectives.

Quantitative analysis of cell morphology was performed using cell profiles as we have done previously.^31,69^ The attached cell number was determined by applying the Otsu adaptive thresholding method on the DAPI channels. The cell morphology was analyzed by applying the Otsu adaptive thresholding method on both DAPI and phalloidin channels. The parameters describing cell morphology were quantified in terms of the cell spreading area (the number of pixels occupied) and form factors (numbers closer to 1 describe rounder cells)

### 5.6 Cell Focal Adhesion on Films

Cell focal adhesion was also analyzed using immunohistochemical staining. hMSCs were seeded onto MSN films at a density of 1500 cells cm^–2^. Briefly, after 5 days of culture, hMSCs were fixed with 4% PFA for 10–15 min, permeabilized with Triton X-100 (0.2% (vol/vol) in PBS) for 10 min, and blocked with blocking buffer (4% (w/v) BSA and 0.05% (v/v) Tween in PBS) for 1 h at RT. After that, cells were incubated with the Alexa Fluor 647 Anti-Vinculin antibody (1:200 in blocking buffer) overnight at 4 °C, followed by washing three times with PBS. To visualize actin bundles and nuclei, hMSCs were stained with Alexa Fluor 488 Phalloidin (1:40 in PBS) for 1 h and DAPI (1:100 in PBS) for 15 min at RT. After gently rinsing with PBS, samples were mounted Dako and imaged with a Nikon Eclipse Ti-E microscope (Nikon Instruments Europe BV, the Netherlands) using a 40× objective. Images were further processed to assess the length and area of vinculin using NIS-Elements AR Analysis 5.30 with a custom-made pipeline.

### 5.7 Computer Simulations

We used Python 3.8 to create a 2D grid (2000 × 2000) on which we could place circles with a diameter of 70 nm to represent MSN particles coating a 2D surface. RGD-containing MSN particles were distributed on the surface randomly among other MSN particles using the function “random.sample” from the Python random module. For each simulation, we repeated the random coatings a hundred times in order to avoid any sampling bias. Using the coating simulations, we aimed to answer two main questions:

(1) What percent of MSN-RGD particles need to be used in order to obtain sufficient aggregation between RGD particles to provide the basis for optimal focal adhesion formation?
(2) What is the main difference between low- and high-local-density MSN-RGD particles in terms of the RGD spacing and particle aggregation?

For the first question, we simulated surface coatings for 10, 25, 50, and 75% of MSN-RGD particles. A representative example of nanoparticle random distribution on surfaces is shown in Figure 2a. Note that we use a mean-field approximation on the MSN nanoparticles. Any RGD ligand that is attached to the MSN has no specific location—the dimensions of the nanoparticle itself are invalid. We defined MSN-RGD particles as “neighbors” if they were at most 70 nm apart from one another (surface-to-surface Euclidean distance). We then reported the mean ratio of MSN-RGD particles with at least one neighbor over the total number of MSN-RGD particles for each wt % composition. We also reported the average number of neighbors per MSN-RGD particle (average of 100 coatings) in each wt % to provide an idea of the aggregate size of the RGD peptides in each setup. Additionally, we investigated the effect of the nanoparticle size on RGD ligand distribution at a certain RGD global density (25% RGD). Same as calculating the number of the “neighbors,” we adjusted the diameter of the circles, which in turn recalculated the position of each MSN nanoparticle and how many nanoparticles could fit within a 2000 nm × 2000 nm surface. We then placed RGD particles at random locations until a global density of 25% was achieved. We then identified RGD-MSN particles that had an RGD-MSN neighbor within the cutoff distance of 70 nm.

For the second question, we introduced a metric called the effective distance, which indicates the average distance between any two MSN-RGD particles on the 2D surface. Biologically, the effective distance corresponds to the distance a cell needs to span in order to adhere to any two RGD-carrying particles. To simulate the high-local-density MSN-RGD particles, we assumed they carried twice as many RGD peptides as the low-local-density MSN-RGD particles did. The average distance between any RGD particles is then calculated as the sum of all distances between RGD ligands (Figure 5a). For the high-density clustering, due to mean-field approximation, we assume that the distance between two RGD nanoparticles on the same MSN particle can be safely neglected. Again, due to mean-field approximation, the distance between any two ligands on two different MSN is always the same. We then compared how the effective distance changes between the low- and high-local-density setups. We repeated these simulations for varying surface sizes in order to make sure the results were not affected by the choice of surface size.

### 5.8 Alkaline Phosphatase Assay

Osteogenic differentiation of hMSCs was evaluated by measuring ALP levels after 14 days of culture using an alkaline phosphatase kit (Abcam) according to the manufacturer’s instructions. CyQuant cell proliferation was used to determine DNA content for the normalization of ALP levels. hMSCs were seeded onto MSN films at a density of 4000 cells cm^–2^. For cell seeding, 250 μL of the cell suspension was carefully pipetted on the films or uncoated glass coverslips (negative control), and cells were left to adhere for 4 h. After 4 h of incubation, the cells were refreshed with 2 mL of basic medium or osteogenic medium (basic medium supplemented with 10 nM dexamethasone). After 14 days of culture, cells were harvested from the films or uncoated glass, rinsed with PBS, and then divided into two portions. One portion was used to measure ALP levels, and another one was used to measure DNA content.

To measure ALP levels, cells were resuspended in appropriate volumes of assay buffer provided in the kit. Then, the samples were incubated with the MUP substrate (5 mM) at 25 °C for 30 min in the dark. After that, a stop solution was added to the samples, and the fluorescent signal was measured on a spectrophotometer at 360 nm. ALP values were normalized with total DNA content per sample and expressed as an *x*-fold increase compared to the negative control.

To measure DNA content, cells were frozen-thawed for three cycles at −80 °C and then digested by incubating with a proteinase K solution (1 mg/mL in Tri-EDTA buffer, pH 8.0) overnight at 56 °C. After another three cycles of freezing-thawing at −80 °C, the proteinase K-digested samples were lysed with RNAse-containing lysis buffer for 1 h at RT. Afterward, the cell lysate was mixed with a GR-dye solution (provided in the CyQuant kit, 1:200 in lysis buffer). After 15 min of incubation, the fluorescent signal was measured with a spectrophotometer at λ_ex_ = 485 ± 10 nm and λ_em_ = 530 ± 20 nm. Absolute DNA amounts were calculated using the standard curve prepared following the supplier’s instructions.

### 5.9 Statistical Analysis

All data were statistically analyzed using one-way analysis of variance (ANOVA), followed by Tukey’s multiple comparison post-hoc test. All data were expressed as the mean ± standard division. For all figures, the following *p*-values apply: *p < 0.05; **p < 0.01; ***p < 0.001. A difference with a *p*-value less than 0.05 was considered statistically significant.

## Supporting Information Available

Characterization of amine-modified MSN using fluorescent labeling; optical images of MSN films; SEM images showing the surface structure of the MSN films; 3D laser scanning microscopy data of the surface profile of the MSN films; water contact angle of glass substrates and different MSN films; schematic illustration showing four examples of random nanoparticle distribution on the 10% MSN-RGD_H_ surface; computational description of how the size of the nanoparticle influences the ratio of MSN-RGD particles; effect of RGD global density on hMSC attachment; and additional details on MSN synthesis and characterization (PDF)
