# Supplementary material for: Development of Mesoporous Silica Nanoparticle-Based Films with Tunable Arginine–Glycine–Aspartate Peptide Global Density and Clustering Levels to Study Stem Cell Adhesion and Differentiation
Source: ACS Appl Mater Interfaces. 2023 Aug 1;15(32):38171–84. doi: 10.1021/acsami.3c04249 (PMC10436245; doi:10.1021/acsami.3c04249)
Supplement: Supplementary file 1 — am3c04249_si_001.pdf [file am3c04249_si_001.pdf]

Supporting Information

Development of Mesoporous Silica Nanoparticle Based Films with Tunable Arginine-Glycine-Aspartate Peptides Global Density and Clustering Levels to Study Stem Cell Adhesion and Differentiation

*Xingzhen Zhang, Zeynep Karagöz, , Sangita Swapnasrita, Pamela Habibovic, Aurélie Carlier, and Sabine van Rijt**

* Corresponding author Dr. Sabine van Rijt
Department of Instructive Biomaterials Engineering
MERLN Institute for Technology-Inspired Regenerative Medicine, Maastricht University
P.O. Box 616, 6200 MD Maastricht, the Netherlands
E-mail: s.vanrijt@maastrichtuniversity.nl

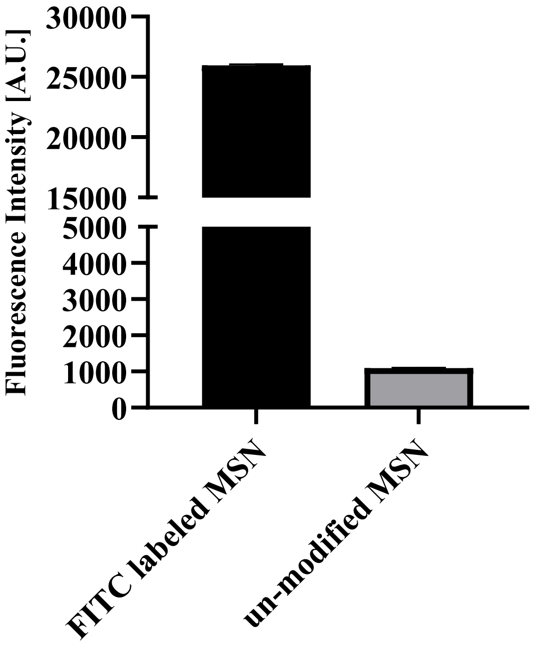

Figure S1. Fluorescence intensity (a.u.) of FITC-NHS labeled MSN_{NH2} Un-modified MSN used as control.

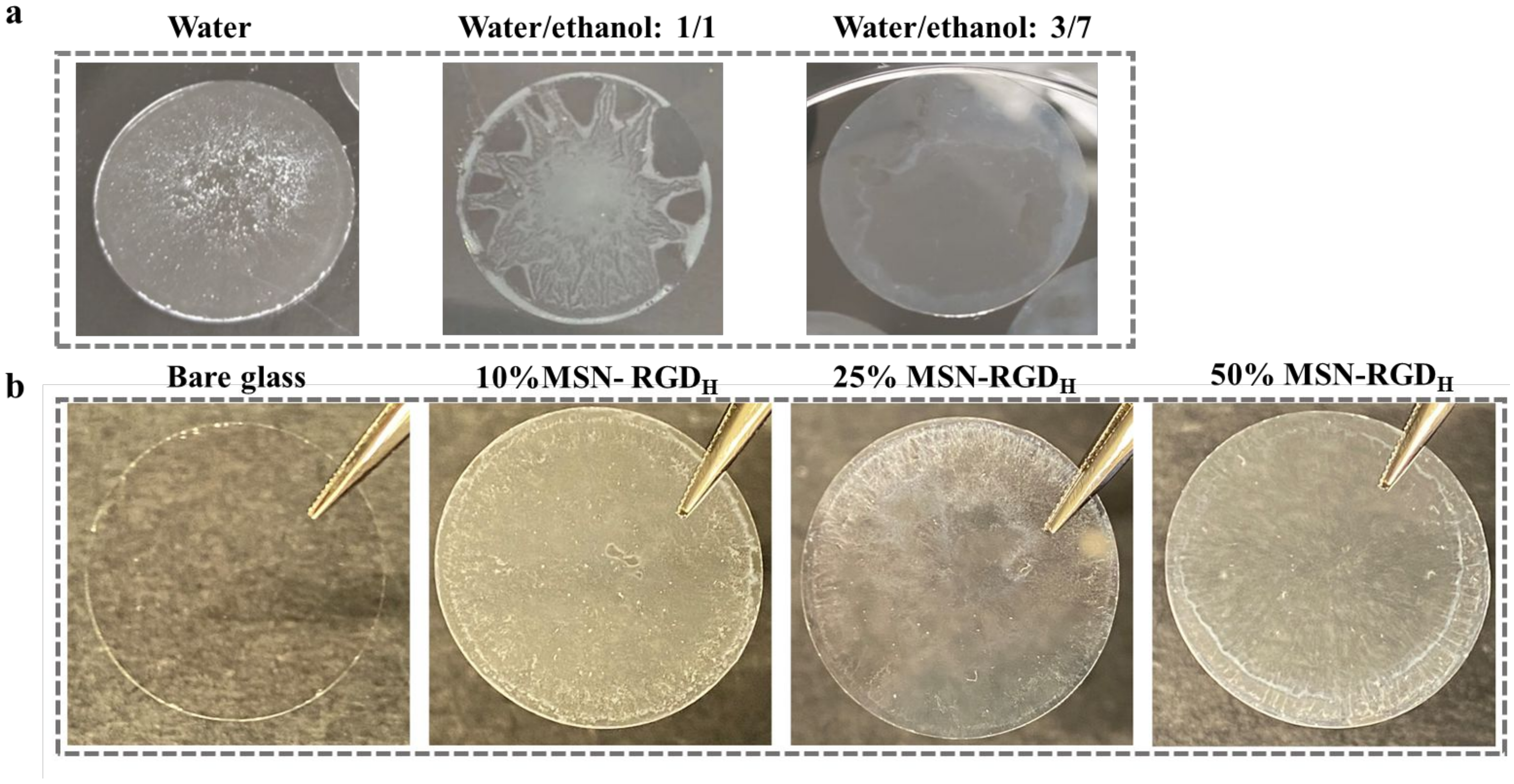

Figure S2. Optical image of MSN films. a) The effect of different ratios of ethanol/water as MSN-RGD_H dispersion solvent on spin coating quality. b) Optimized and homogenous surface coating of MSN surface obtained from different ratios of MSN-PEG and MSN-RGD_H.

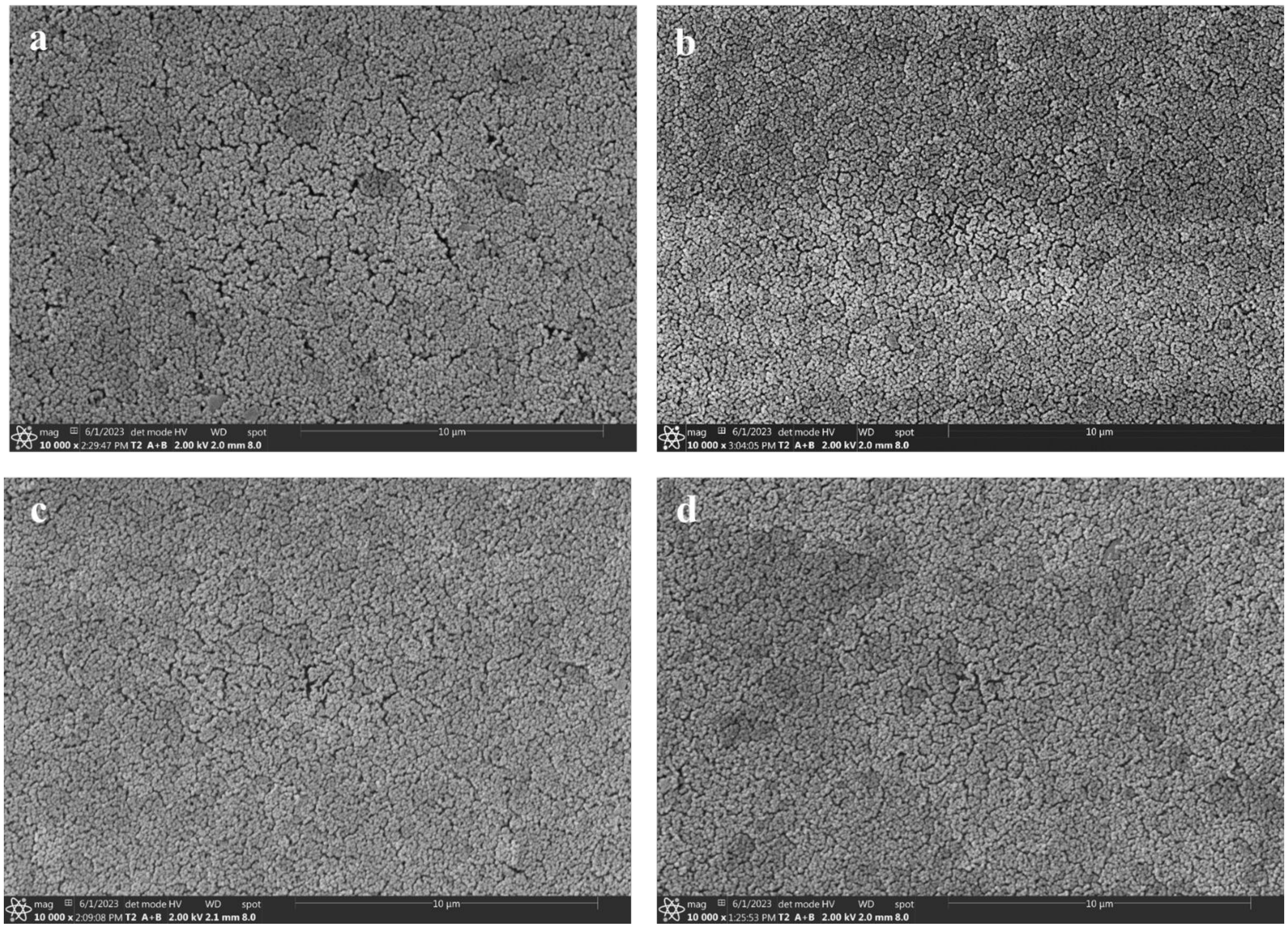

Figure S3. SEM images showing the homogenous surface structure of the MSN films made from a) 50% MSN-RGD$_L$, b) 25% MSN-RGD$_H$, c) 100% MSN-RGD$_L$, and d) 50% MSN-RGD$_H$ nanoparticle compositions.

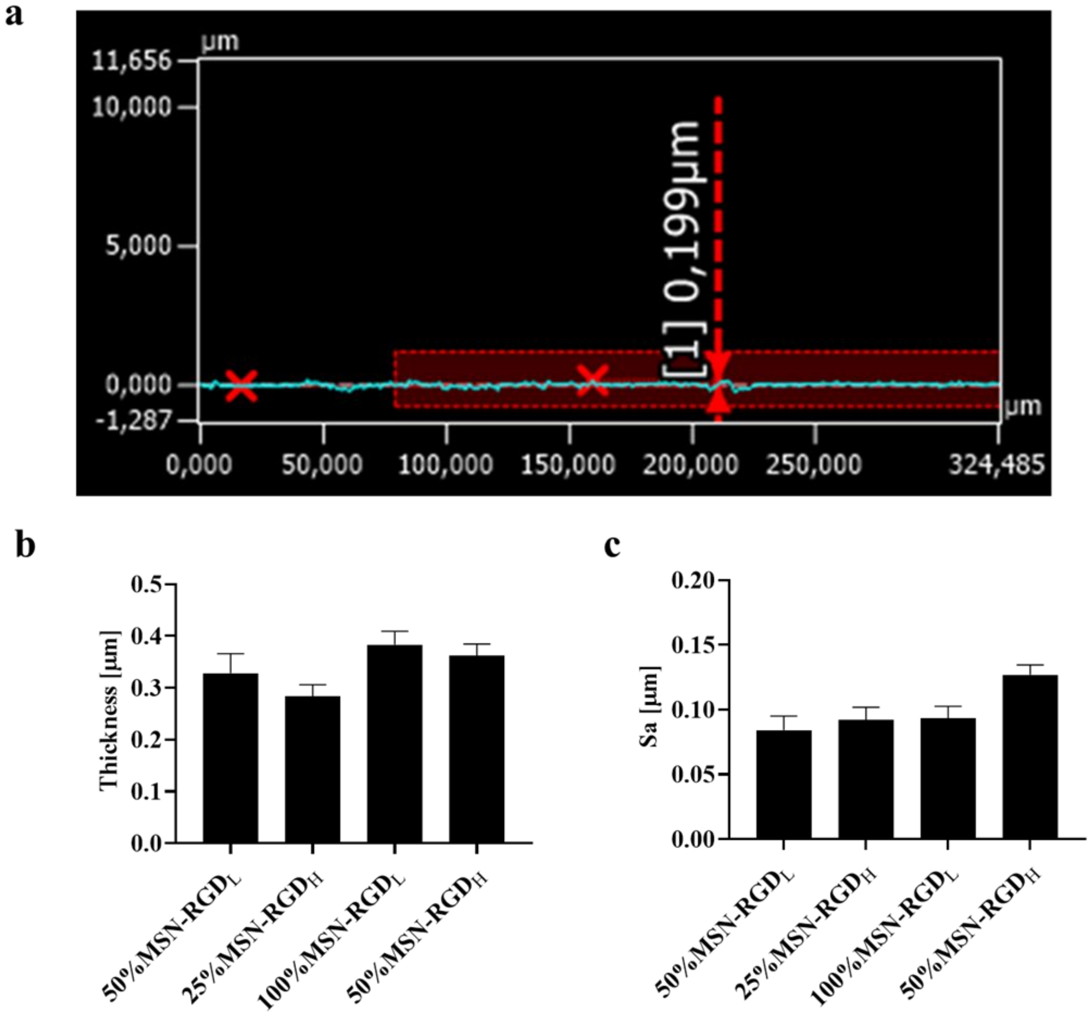

Figure S4. a) A representative 3D laser scanning microscopy image showing the smooth surface profile of the MSN film. b) Surface thickness and c) surface roughness.

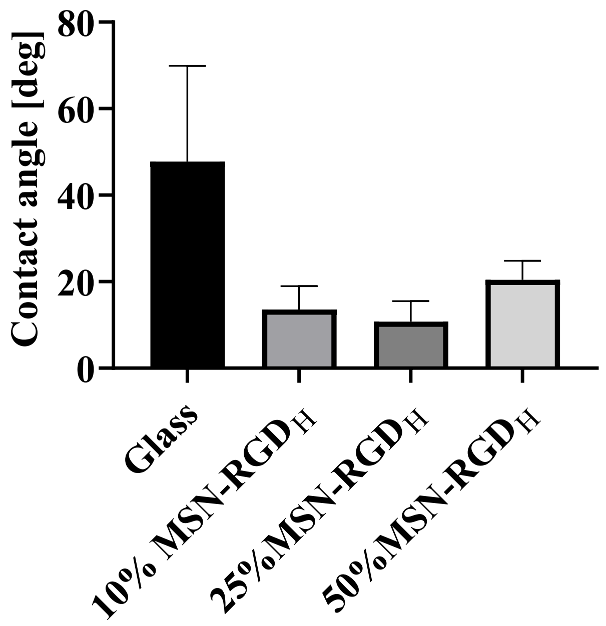

Figure S5. The water contact angle of glass substrates and different MSN films.

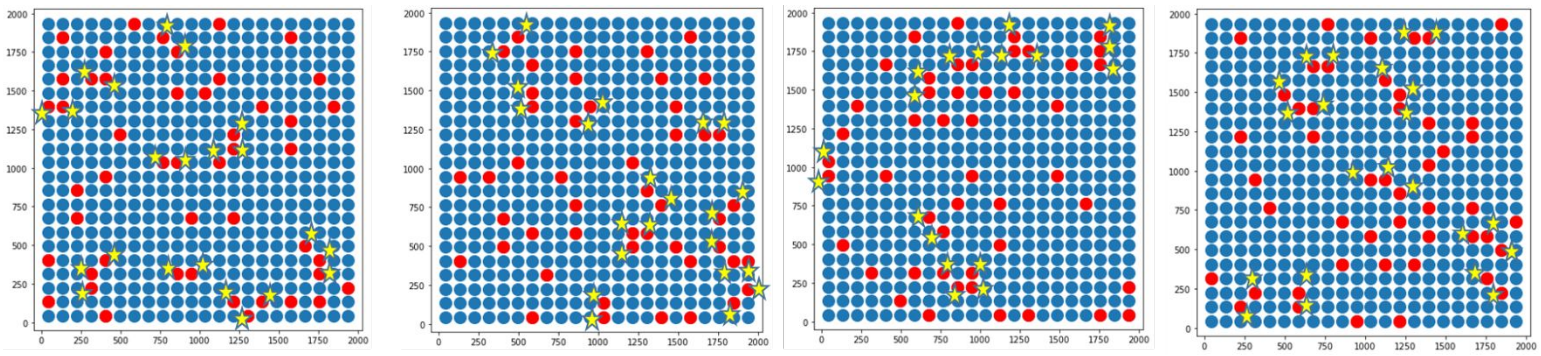

Figure S6. A schematic illustration showing four examples of random nanoparticle distribution on 10% MSN-RGD_H surface, 484 particles in total, 48 of them are MSN-RGD_H in red. The yellow stars indicate the RGD particles having another neighboring RGD particle within 70 nm distance.

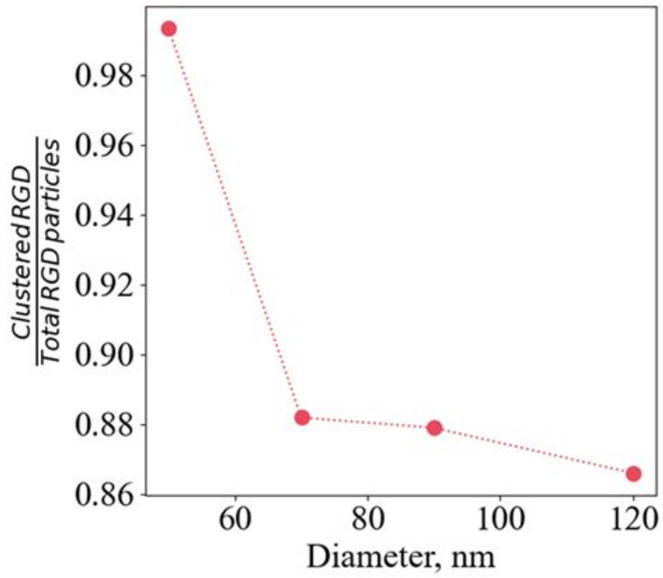

Figure **S7.** Computational description of how the size of the nanoparticle influences the ratio of MSN-RGD particles which have at least one other MSN-RGD within 70 nm distance (clustered RGD) over the total number of MSN-RGD particles on the surface.

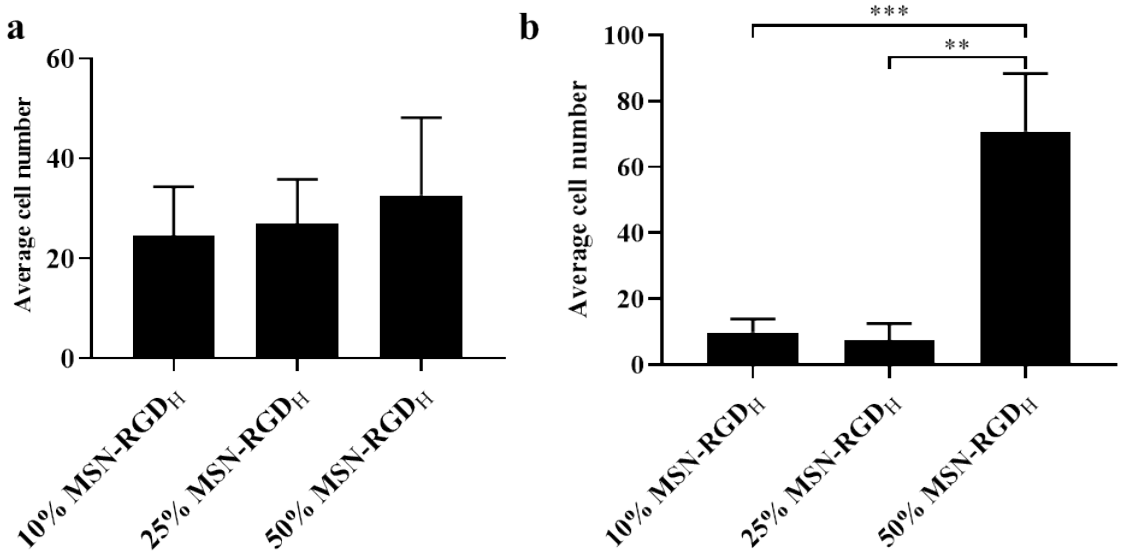

Figure S8. Effect of RGD global density on hMSC attachment. Quantification of attached cell number of hMSCs cultured on 10% MSM-RGD_H, 25% MSM-RGD_H and 50% MSM-RGD_H films for a) 1 day and b) 3 days. Data are expressed as the mean ± SD (n=3). *p < 0.05; **p < 0.01; ***p < 0.001.

Synthesis and characterization of MSN_{NH2}

To synthesize *MSN_{NH2}*, a mixture of 1.73 g tetraethyl orthosilicate (TEOS) and 14.3 g triethanolamine (TEA) was heated to 90 °C under static conditions for 20 min (Solution 1). Solution 2 was prepared by adding100 mg of ammonium fluoride (NH4F), 2.41 ml of cetyltrimethylammonium chloride (CTAC) to 22 ml of bi-distilled water (DIW) and heated to 60 °C for 10 min. Next, Solution 2 was quickly added to solution 1, and the mixture was stirred vigorously for 20 min, after which 138.2 mg TEOS was added to the mixture in four equal portion every 3 min, and stirred for 30 min. In the next, a mixture of 19.3 mg TEOS with either 20.5 mg 3-Aminopropyl triethoxysilane (APTES, in the case of MSN-NH2) or 20.5 mg 3-mercaptopropyl triethylsilane (MPTES, in the case of MSN-SH) was added and stirred overnight at room temperature. The following day, particles were collected by centrifugation and washed once with ethanol, and then redispersed in an

ethanolic ammonium nitrate solution. Then, the mixture was refluxed at 90°C for 45 min. Afterwards, MSN were collected by centrifugation, washed once with ethanol, redispersed in 100 ml of a hydrochloric acid solution (HCl 37% in DIW), and refluxed again at 90°C for 45 min. Finally, MSN were collected, re-dissolved in ethanol and kept at -20°C for future use.
